# Flowability and Strength Characteristics of Binary Cementitious Systems Containing Silica Fume, Fly Ash, Metakaolin, and Glass Cullet Powder

**DOI:** 10.3390/ma16196436

**Published:** 2023-09-27

**Authors:** Mohammad Iqbal Khan, Yassir M. Abbas, Galal Fares, Fahad K. Alqahtani

**Affiliations:** Department of Civil Engineering, College of Engineering, King Saud University, P.O. Box 800, Riyadh 11421, Saudi Arabiagalfares@ksu.edu.sa (G.F.); bfahad@ksu.edu.sa (F.K.A.)

**Keywords:** binary mixes, flowability, strength, heat of hydration, pore system, supplementary cementitious materials

## Abstract

The present study examines the effects of supplementary cementitious materials (SCMs) on the flowability and strength development of binary mixes. This study was primarily motivated by the need to bridge the knowledge gap regarding paste and mortar mixes containing binary cement from a variety of performance perspectives. This study examined the flowability and strength development of binary mixes in their pastes and mortars when they contain various doses of silica fume (SF), fly ash (FA), metakaolin (MK), and glass cullet powder (GP) compared with the control mix. While the presence of SF and MK reduced workability because of the nature of their particles, the addition of FA and GP improved it to a certain extent because of the spherical and glassy nature of their particles, respectively. In addition, GP was used to compare its performance against SF, MK, and FA as an alternative cementitious material. In this study, the GP performed comparably to the other SCMs investigated and was found to be satisfactory. An investigation of the rheological properties, heat of hydration, thermal analysis, and pore systems of these mixes was conducted. Compared to the control mix, the presence of 5% GP improved the rheological properties and reduced the heat of hydration by 10%. The reduced workability in SF and MK mixes resulted in a lower content of pore water, while GP and FA incorporation enhanced it, owing to improved workability. The pore area is related to the pore water, which is directly related to improved workability. According to the following order, SF > MK > GP > FA, the strength was highest for mixes containing SF and MK, whereas, with GP and FA, there was a gradual reduction in the strength proportional to replacement level and improved workability. SF, GP, and FA can be identified as performance enhancers when formulating ternary and quaternary cementitious systems for low-carbon cement.

## 1. Introduction

### 1.1. Background

A robust application of sustainability theory has been made in recent years by using industrial by-products as mineral admixtures for cement-based materials, which has been verified as a reliable characteristic of sustainable development [1,2,3]. It is generally known that cement-based materials are composed of three basic components: cement, water, and aggregates [4,5,6]. In addition to these three substances, there are usually other substances that make up the cementitious system. A variety of mineral admixtures are used in the manufacture of these concretes, including fly ash (FA), silica fume (SF), and metakaolin (MK).

The ternary combination of FA and both SF and MK is well documented [7,8,9]. However, the use of a normal mixing process is common practice for the production of concrete containing different combinations.

### 1.2. Literature Survey

In the literature, it has been shown that mineral admixtures likely improve the mechanical properties of the mixture by virtue of their pozzolanic or self-cementitious nature [10,11,12]. A mineral admixture can reduce the cost of a concrete mixture as well as make it more workable [13,14,15]. Furthermore, fresh concrete mixtures that contain mineral admixtures tend to have lower bleeding rates [1]. In addition, the resistance of the mix to freeze-thaw cycles [16], sulfate attack [17], acid attack [18], alkali–aggregate reaction [19], and reinforcement corrosion [20] was reported to be improved with the addition of mineral admixtures. Additionally, the reduction of water infiltration speed was mentioned as an improvement with the addition of mineral admixtures [21,22]. Nevertheless, ultrafine mineral admixtures, like SF and MK, were found to significantly enhance the above-mentioned properties of the cementitious systems [23,24,25].

The properties of SCMs with mineral admixtures were also studied by Güneyisi and Gesolu [26]. The study examined cementitious blends that contained Portland cement (PC), metakaolin (MK), and fly ash (FA) mixed in binary and ternary proportions. Researchers have demonstrated that when FA and MK are added to ternary blends, fresh characteristics and rheology are significantly improved over binary blends of either FA or MK alone. The cenospheres in FA are responsible for the enhancement in workability because of the rolling-over effect, while the flaky, angular, and porous structure of MK particles is responsible for the reduction in workability. The optimized blend of FA and MK compensates for the drawback of MK on workability while gaining its initial effect on strength. Benli et al. [27] conducted an experiment by immersing granulated SF and a ternary mixture of FA without seawater and 10% by weight of MgSO_4_ solution in self-compacting mortars containing binary and ternary mixtures. The researchers found that all binaries and ternaries of SCMs, as well as control specimens, showed enhanced compressive and tensile strength up to 90 days before this strength began to decline at 180 days. Similarly, Meng et al. [28] investigated how the additions of a conventional Portland cement paste dispersion, SF, and SF and PCE combined affect the properties of the cement paste when it is fresh and hardened. The results of this study indicated that SF accelerates the hydration of cement and reduces the time needed for cement to set in relation to the SF content within the paste when it is present at an early stage in the process.

In addition, Snehal et al. [29] reported on the effects of nano-silica on the hydration characteristics of binary, ternary, and quaternary blended cement pastes and mortars that contain micro- to nano-sized admixtures. There were three different types of admixtures used in the mix: fly ash, ultrafine FA (UFFA), and colloidal nano-silica (CNS). Based on the experimental results, it was found that a CNS dosage of 3% is the optimal CNS dosage for binary blended cement composites. Furthermore, Han et al. [30] synthesized binary, ternary, and quaternary mixtures using limestone (LS) powder (0–10%), calcined Hwangtoh clay (0–20%), and granulated blast furnace slag (0–30%). Based on the findings of the study, the researchers concluded that the quaternary mixed paste they developed during their study exhibited good durability and sustainability, which makes it an excellent material for a variety of applications. Additionally, Han et al. [31] developed binary, ternary, and quaternary pastes with LS powder (0–10%), MK (0–15%), and FA (0–30%). The study concluded that the paste that was blended with quaternary particles had excellent durability and sustainability, as well as a promising future for further advancement.

Likewise, Lin et al. [32] conducted an experiment and designed a response surface model to optimize quaternary composite pastes containing LS, MK, and FA. In the study, mineral admixtures decreased flow, compressive strength, hydration heat, and CO_2_ emissions; however, the electrical resistivity increased exponentially as LS and MK increased. The use of SF and ground granulated blast furnace slag (GBFS) as the base material for SCMs was also evaluated by Yön et al. [33] for their abrasion and high-temperature resistance. Based on their experiments, SCM mixes containing SF showed a reduction in flowability with an increasing SF rate compared to the control, whereas SCM mixes incorporating GBFS showed an increase in flowability with an increasing GBFS dosage. Furthermore, several formulations of alkali-activated cement (AAC) and binary and ternary cement were also recently studied with superplasticizers by de Hita and Criado [34] to improve their properties. According to their experimental results, the plasticizing effect of composite cement is independent of the type of addition (slag or FA), and it remains constant for at least two hours after mixing. However, when using AAC, this effect decreases rapidly, probably because the additives are not chemically stable in alkaline media. The main aim of the current study is to pave the way for a comprehensive study of gradual improvement until reaching a low-carbon concrete with the minimum footprint. In this study, the binary systems of different supplementary cementitious materials are covered, while the ternary and quaternary systems formulated from these conventional supplementary cementitious materials, as well as non-conventional ones, will be further investigated later.

### 1.3. Importance and Aim of the Study

According to the literature review, several studies have examined the effects of mineral admixtures on cement-based materials. However, relatively little information is available on the flowability and strength development of paste and mortar mixes containing binary cement from different performance points of view. In this study, we investigate the impact of cement-based materials containing various SF, FA, and MK dosages on the flowability and strength development of binary mixes in their pastes and mortars compared to the control mix as the initial step required to formulate ternary and quaternary mixes necessary for low-carbon concrete. This study provides a thorough analysis of various SCMs and their effects on binary mixes, revealing optimization strategies for better flowability and strength. Individual SCMs, like SF, FA, MK, and GP, contribute uniquely, from refining pore structures to promoting sustainability.

## 2. Materials and Methods

### 2.1. Materials

**Figure 1:**
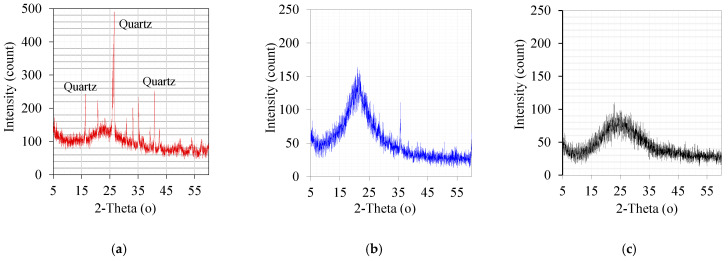
X-ray diffraction patterns of (**a**) FA, (**b**) condensed SF, and (**c**) GP.

Different conventional and alternative supplementary cementitious materials were used in this study. Silica fume, class F FA, MK, and ground glass cullet (GP) were procured and analyzed using different techniques. Their chemical and physical properties are shown in Table 1. The main fine aggregate used in this study is identified as dune red sand (RS). The X-ray diffraction (XRD) analysis of fine powder is shown in Figure 1. The characteristic amorphous structures of SF and GP are confirmed in the figure. Both FA and MK are composed of a combination of amorphous and crystalline silicate structures. They have similar structures; accordingly, the XRD pattern of FA is given in the figure. Table 2 displays the physical characteristics in accordance with ASTM C128 [35]. The median particle sizes of all powders (D50) are shown in Table 1. Chemical mineralogical analyses of fine powders were conducted on X-ray fluorescence, model AXIOS mAX, and X-ray diffraction machines from PANalytical (Almelo, The Netherlands). The particle size distribution analysis was conducted on a laser particle size analyzer, model LA-950V2 (Horiba, Kyoto, Japan).

### 2.2. Particle-Size Distribution

**Figure 2:**
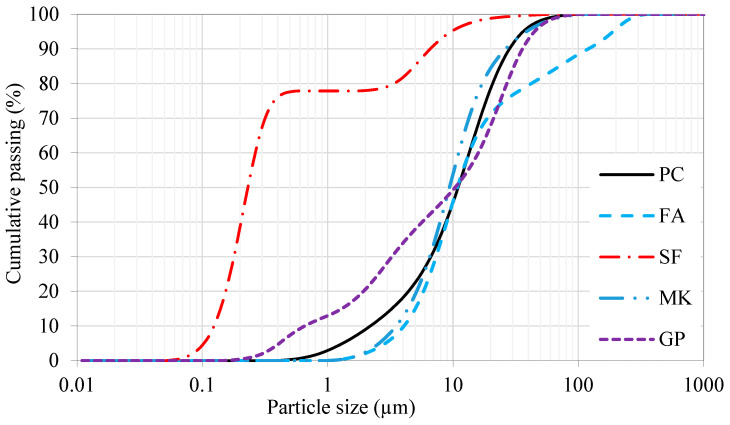
Laser particle-size distribution analysis of fine powders.

**Figure 3:**
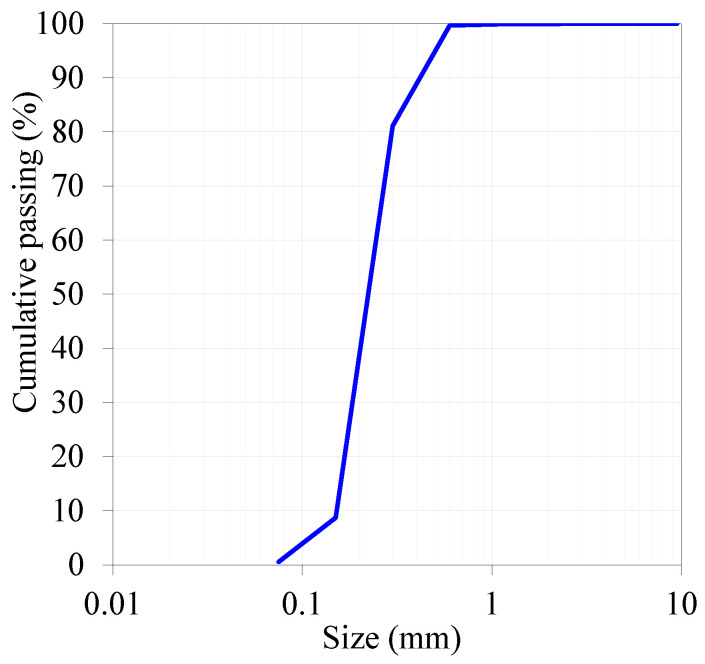
Sieve analysis of local fine aggregate (RS).

Figure 2 and Figure 3 show the particle-size distribution of fine powder and fine aggregate, respectively. Figure 2 demonstrates that silica fume is the finest among the fine powders, followed by GP and MK, while FA and cement have similar particle distributions. It is also evident from the figure that the GP powder contains fractions of nanoparticles (10%) in the range of 200–700 nm. The sieve analysis of the red dune sand collected from the Arabian Peninsula desert has a median particle size of about 150 μm.

### 2.3. Testing Scheme

In this study, paste and mortar mixes were prepared using a water-to-binder ratio of 0.35, a sand-to-binder ratio of 1.5, and a maximum replacement level of 25%, as in the case with GP and FA. The mini-slump flows of different mixes under the effect of a poly-carboxylate ether-based superplasticizer dosage of 0.22% (percentage of solid content per binder), as per ASTM C109 [36], were determined. The initial flow of the control mix was adjusted to a flow value of about 220 ± 20 mm. Then, the effect of each replacement level would be apparent and easily evaluated. Three initial replacement levels of 5, 10, and 15% were selected to establish a trend. The type of SCM that increases workability was further evaluated at a higher dosage of 25%. The composition of the mixes is listed in Table 3. In the min-slump flow test, a truncated cone with a top diameter of 70 mm and a base diameter of 100 mm was applied with 15 flow table drops to measure the final flowability diameter. The compositions of the mixes are shown in Table 3. The differences in SCM additive amounts, specifically GP and MK, were intentional based on trial mixes. It was found that GP could be used up to 25% because of its unique properties, while MK’s reactivity was limited to 15% to ensure the mix quality. It is noteworthy that the focus of the current investigation was on illustrating the varied effects of SCM concentrations rather than making direct comparisons. The compressive strength of the samples was measured as per ASTM C109 using a ToniTech universal compression testing machine (capacity 3000 kN). Three replicate samples were used in this study.

Selected paste samples were prepared for various purposes. The first purpose was to test for rheological properties using an Ofite viscometer, model 900 from Ofite (Houston, TX, USA). The second one was for the evaluation of the heat of hydration using an isothermal calorimeter, model TAM AIR 90 C230 from TAM AIR Instruments (New Castle, DE, USA). The third one was for the evaluation of the pore distribution system using mercury intrusion porosimetry (MIP), model AutoPore IV 5900 from Micromeritics (Norcross, GA, USA). MIP analysis was conducted on pieces taken from tested samples and stored in isopropanol-2 alcohol for 24 h, then dried in an oven at 95 ± 5 °C for 24 h. The average sample mass of about 1 g was used in this test. The last purpose was to quantify different cementitious phases after hydration using a TG analyzer, model SDTQ600 from TAM AIR Instruments (New Castle, DE, USA). Under similar conditions to those used in the preparation of samples for MIP, pieces were ground using a mortar and pestle inside an isolated desktop chamber. A powder of about 15 g was used in this analysis under nitrogen gas conditions (flow of 50 mL/min) and a temperature incremental ramp rate of 10 °C/min.

## 3. Results and Discussion

### 3.1. Paste Mixes

#### 3.1.1. GP Role and Effect on Rheology

**Figure 4:**
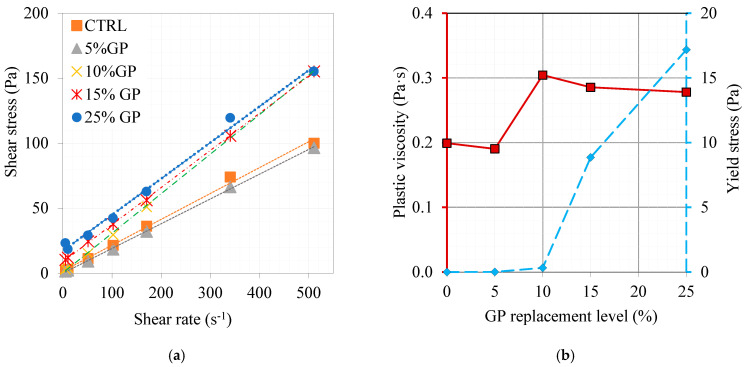
Effect of GP content on the development of plastic viscosity and yield stress in paste mixes. (**a**) shear stress/shear rate relationship and (**b**) plastic viscosity and yield stress as a function of GP replacement level.

The rheological measurement is another tool to evaluate the effect of different replacement levels and various types of cementitious materials on the rheological properties. The GP, which has been shown to have nano-sized particles, was tested in paste mixes with the same components shown in Table 3 for rheological properties. The effect of the GP replacement level on the rheological properties is shown in Figure 4a. The extracted rheological parameters are shown in Figure 4b. The presence of 5% GP caused a significant reduction in the plastic viscosity, which could be attributed to the slippery effect of its glassy particles, as previously reported [37]. The known glassy nature of the glass powder was reflected in the performance of its particles. This glassy nature played an improving role in the slippage of the whole cementitious system in addition to its negligible demand for water since its glassy nature inhibits any water demand. However, as the GP replacement level increased, the other side effect of its angular particles becoming crowded, regardless of their glassy nature, caused this remarkable increase in plastic viscosity. However, as the content increased, the number of nano-sized particles increased significantly in a way that initially increased the plastic viscosity, followed by a gradual reduction in the plastic viscosity and a significant increase in the yield stress.

#### 3.1.2. Comparison between GP and Other Cementitious Materials

##### Rheological Properties

**Figure 5:**
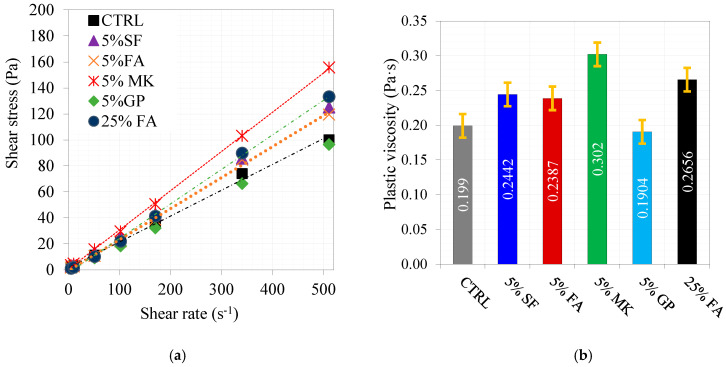
Rheology of selected cement pastes—(**a**) shear rate versus shear stress and (**b**) plastic viscosity of each paste mix.

The rheology of the selected mixes, based on their reported performance, is shown in Figure 5. Compared to the control mix, the presence of cementitious materials led to a substantial increase in the plastic viscosity, except with GP. It is evident that the plastic viscosity reached its maximum value with a replacement level of 5% MK, while the lowest value was obtained with a replacement level of 5% GP. This was attributed to the nature of the particles in each type of cementitious material. As 5% GP delivered different behaviors, special attention was given to GP as an alternative cementitious material. The nano-sized SF particle and the highly pozzolanic nature of its particles increased the adhesion and physical attraction of electrostatic forces among all particles, which caused the reported increase in the plastic viscosity compared to the control mix. Moreover, this can also be justified by the increase in water demand. Similarly, the MK of flaky and porous particles increased the water demand, which caused this remarkable increase in the plastic viscosity under the same replacement level. It can also be explained by the effect of packing density, which is very obvious in the cases of GP and FA [38]. GP had the highest packing density, which reduced the need for water; however, under a fixed amount of water and superplasticizer similar to the control mix, the availability of water increased; consequently, the friction among particles decreased, and the plastic viscosity decreased as well. The packing density was lower than GP in the case of FA. Moreover, the presence of two types of particles, spherical and angular (amorphous), increased the plastic viscosity. Any additional increase in FA caused an obvious increase in plastic viscosity.

##### Heat of Hydration

**Figure 6:**
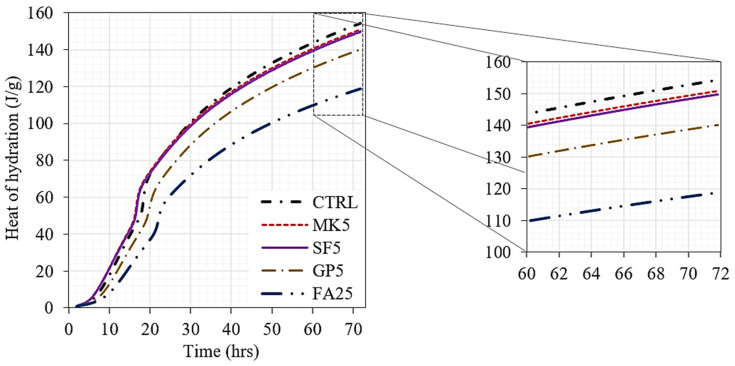
Heat of hydration profiles of different cementitious systems.

The development of the heat of hydration over time until 72 h is presented in Figure 6. The effect of the replacement level on hydration is notable. The cement paste mix with 25% FA had the lowest hydration flow. However, 5% SF and 5% MK provided similar levels of hydration and were higher than those of the mix with 5% GP and the control mix. It is worth noting that at a very low initial hydration time of less than 20 h, 5% SF and 5% MK delivered a high early heat of hydration compared to the control mix. The cumulative heat of hydration until 72 h is reported in Table 4. The incorporation of 5% GP led to a 10% reduction in the heat of hydration, while that due to 25% FA reached 23% less than that of the control, and both 5% SF and 5% MK led to the same reduction in the heat of hydration by about 2.6%. The heat flow measurement was related to the thermal coefficient of the materials and the heat flow through conduction from material to material as a whole, while the temperature measurement was related to the temperature at a certain point. Therefore, GP is the most efficient powder for reducing the heat of hydration.

**Figure 7:**
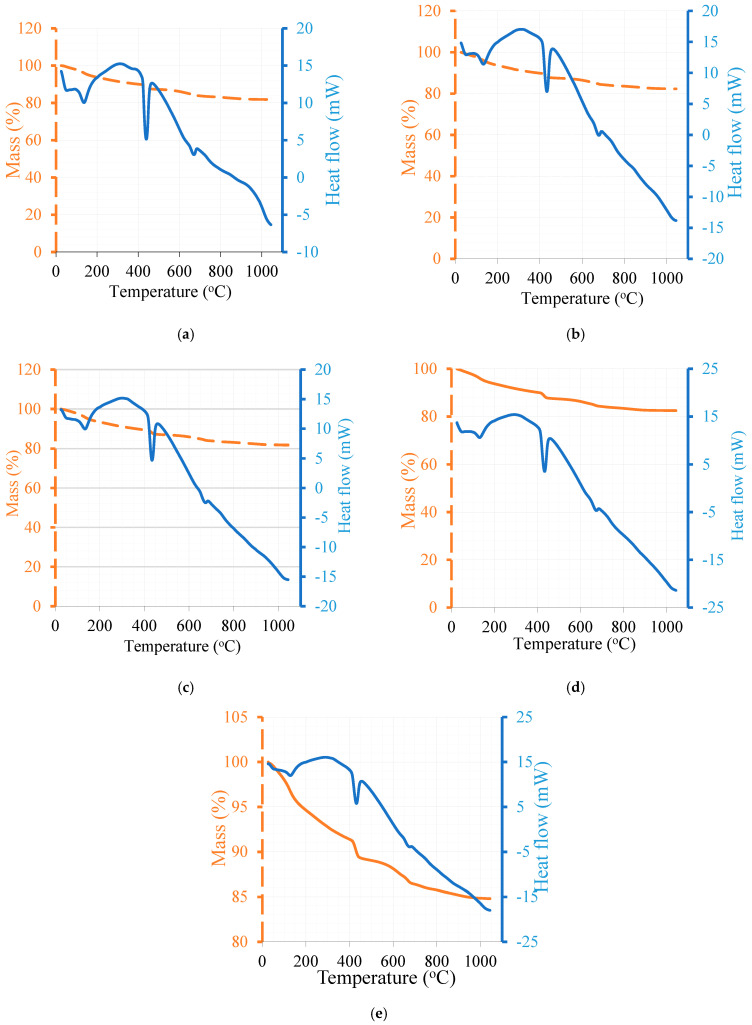
TGA/DSC analyses of different cementitious systems (**a**) CTRL, (**b**) 5% SF, (**c**) 5% MK, (**d**) 5% GP, and (**e**) 25%FA.

The thermal analysis of the pieces extracted from the paste samples used in the measurement of heat hydration is presented in Figure 7. The quantitative data obtained from thermal analysis is presented in Table 5.

By knowing the chemical formula and molecular weight of AFt, portlandite (CH), and calcite (CaCO_3_) and the thermal decomposition reactions, different products can be estimated. In addition, the thermal decomposition of hydrated cement is a dehydration process where water is evolved, and decarbonation takes place when carbonaceous compounds are present where CO_2_ gas is evolved. These reactions in an isolated system are irreversible.

AFt = 3CaO•Al_2_O_3_•3CaSO_4_•32H_2_O (1)

Total molecular weight of H_2_O (**_AFt_**) in AFt = 576

molecular weight of AFT = 1062

AFt = LOI(T_100–200 °C_) × (1062/576)

Portlandite thermal decomposition:

Ca(OH)_2_ -------> CaO + H_2_O (CH = LOI(T_350–450 °C_) × (78/18)) (2)

100     56  18              

Calcite thermal decomposition:

CaCO_3_ -------> CaO + CO_2_ (Calcite = LOI(T_600–750 °C_) × (100/44)) (3)

Once the percentage of water content is known with respect to the total weight of all components, any type of water can be estimated via back-calculation and a conversion factor using the above Formulas (1)–(3). Moreover, water is back-calculated from all hydrated and carbonated phases; accordingly, the combined water can be estimated, and the difference from the total water percentage is estimated as pore water. Similar calculations are shown in detail in [39].

The hydrated lime (CH) calculated in the paste mixes in the temperature range of 350–450 °C shows that 5% GP did not affect the CH content compared to the control mix despite the replacement level of 5%. This result may refer to the acceleration effect of GP powder on cement particles at an early age. On the other hand, the free lime in 5% MK, because of the thermal treatment, contributed to the CH content by an estimated amount of about 1% of the total CH in the mix. The incorporation of 5% SF led to an estimated reduction of about 3.8% in CH compared to the control mix. Moreover, the incorporation of 25% FA did not show a reduction in CH equivalent to the replacement level of 25% but showed an estimated increase in CH of about 1.4%. This increase is again interpreted by the increased number of hydrated cement particles. Normally, in the control mix, the heat of hydration is proportional to the number of cement particles hydrated during the measurement, which can be increased by many factors that accelerate the hydration process, namely, an increased number of cement particles. Ettringite (AFt) formation was estimated in the temperature range of 100–200 °C. AFt formation was found to be proportional to the replacement level in the mix with 5% GP and slightly higher with 5% SF compared to the control mix; however, ettringite formation was the highest in the mix with 5% MK because of its high reactive aluminate phase. It had an ettringite content that was 10% higher than that in the control mix. The ettringite content was the lowest in the mix with 25% FA; however, the content was not equivalent to the replacement level but higher by about 8.7%. This was attributed to the increased number of hydrated cement particles. The estimated pore water in all mixes with powder was higher than that in the control, while it was the highest in the mix with 25% FA followed by 5% GP. The combined water is the highest in the mix with 5% MK and the lowest in the one with 25% FA.

**Figure 8:**
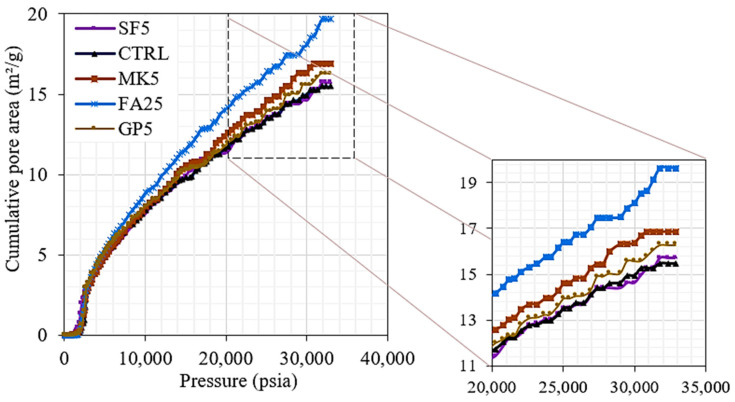
Total pore area as a function of pressure.

**Figure 9:**
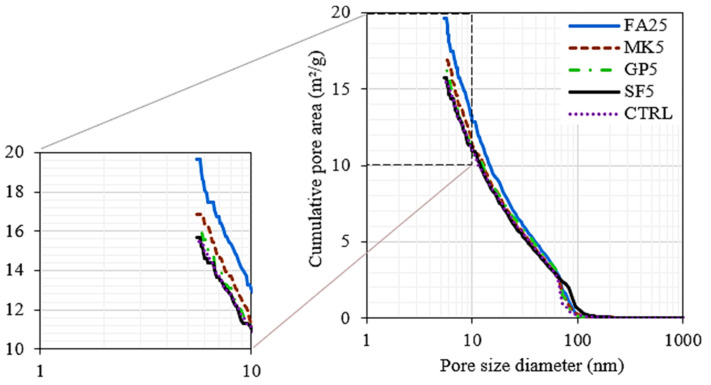
Total pore area as a function of pore size diameter.

The cumulative pore area distribution obtained from the MIP analysis is shown in Figure 8 and Figure 9 and Table 6. It is noted that, in all mixes, the intrusion was negligible until a threshold pressure of about 2000 psi, where the pores larger than 100 nm began filling with mercury. It is noted that the mix with 25% FA, which was shown to have higher pore water, had the highest pore area, which accepted more volume of mercury at the same pressure. The highest pore area in the FA25 mix was then followed by the MK5, GP5, and SF5 mixes, and the control mix had the lowest cumulative pore area. The intrusion data summary of tested paste mixes is shown in Table 6.

### 3.2. Mortar Mixes

#### 3.2.1. Mini-Slump Flow

**Figure 10:**
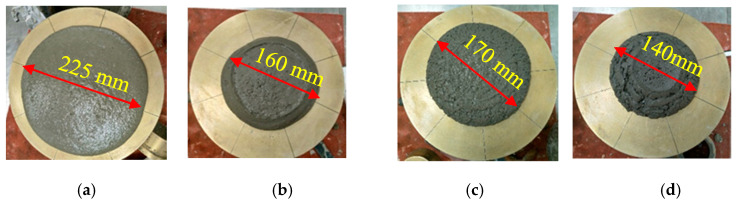
Flowability properties of the mixes containing: (**a**) 0, (**b**) 5, (**c**) 10, (**d**) 15% SF.

**Figure 11:**
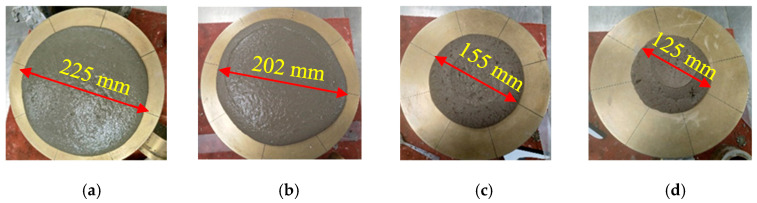
Flowability properties of the mixes containing: (**a**) 0, (**b**) 5, (**c**) 10, and (**d**) 15% MK.

**Figure 12:**
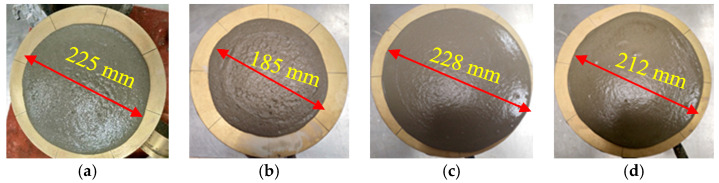
Flowability properties of the mixes containing: (**a**) 0 and 5, (**b**) 10, (**c**) 15, (**d**) 25% GP.

**Figure 13:**
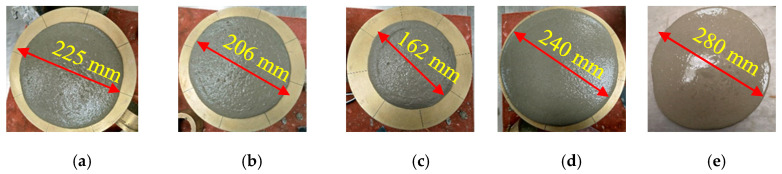
Flowability properties of the mixes containing: (**a**) 0, (**b**) 5, (**c**) 10, (**d**) 15, and (**e**) 25% FA.

In this part of the study, the flow values were monitored in different mixes with SF, FA, MK, and GP, as depicted in Figure 10, Figure 11, Figure 12 and Figure 13. The incorporation of 0, 5, 10, and 15% SF reduced flowability gradually, as shown in Figure 10. However, the initial drop was substantial. Because of its high content of calcined clayey minerals, the incorporation of 0, 5, 10, and 15% MK led to a continuous reduction in flowability, as demonstrated in Figure 11. The flowability of the mixes with different replacement levels of 0, 5, 10, 15, and 25% GP was not affected compared to the control mix, as shown in Figure 12. It was then followed by a notable reduction with 10% GP, then a significant improvement with 15%, and a slight reduction with 25%. This variation could be attributed to the content of nanoparticles in GP, which was significantly more important than the dilution effect. However, the dilution effect became important in the case with 15% GP, and the flowability increased with 25% GP. At this stage, the mix became sticky, and flowability was affected by the yield stress, as previously demonstrated in Figure 4b. The incorporation of a high percentage of FA increased flowability in general, as depicted in Figure 13. However, the incorporation of 5 and 10% FA reduced the workability significantly, and the further incorporation of 15 and 25% increased the workability substantially. This could be justified by the packing density, which reached its maximum effect at the replacement level of 10%, after which the dilution level and the effect of cenospheres become dominant, which improves the workability because of the rollover effect. In general, the net effect of these parameters led to an enhancement of flowability.

**Figure 14:**
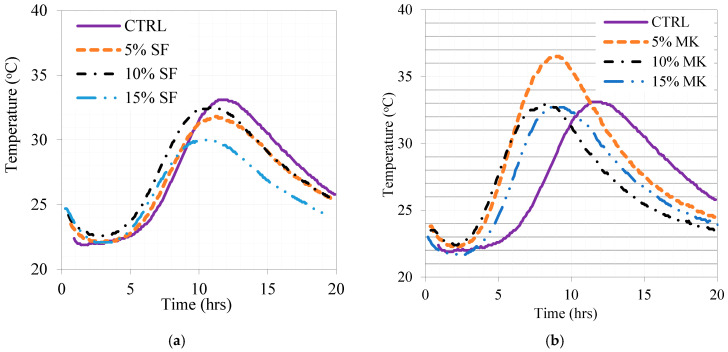

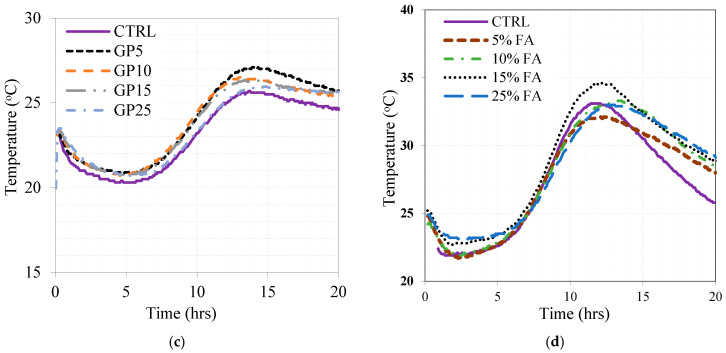
Effect of fine powders and their replacement levels on temperature profiles in (**a**) SF, (**b**) MK, (**c**) GP, and (**d**) FA.

The temperature profiles of the mixes were monitored using the semi-adiabatic technique. The obtained data are shown in Figure 14. Compared to the control mix, the presence of SF shifted the temperature peak to a shorter time as an indication of accelerated hydration. It is evident that the presence of MK led to a significant shift of the temperature peak to a higher temperature, even higher than SF, and shorter time as an indication of its active form. The presence of FA shifted the temperature profile to a lower temperature and a longer time as an indication of the slower pozzolanic activity that directly reduced the heat of hydration. However, the content of 15% FA shifted the peak to a higher temperature compared to the control mix. The effect of the replacement levels of GP powder on the temperature profiles compared to the control and other mixes is shown in Figure 14. It is evident that the 5% GP, followed by 10 and 20%, contributed to the temperature as an indication of its elevated pozzolanic activity. The post-temperature peak behavior is evident for all replacement levels of GP compared to the control mix. All the mixes with GP shifted the temperature peaks to higher values than that of the control mix, which had an insignificant shift to a shorter time. This note refers to a special type of pozzolanic reaction with GP due to the presence of nano-sized particles.

#### 3.2.2. Compressive Strength

**Figure 15:**
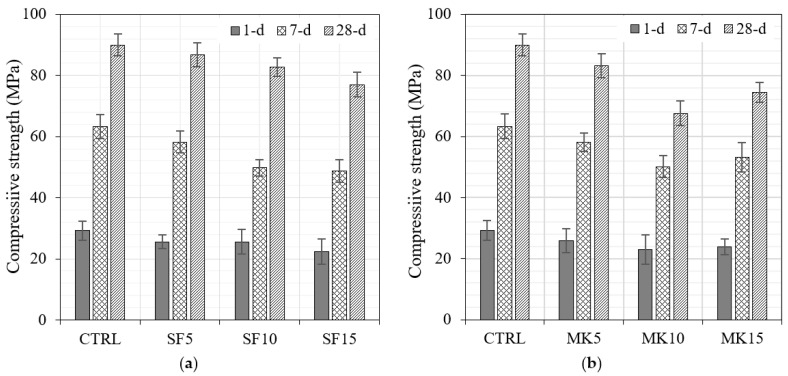

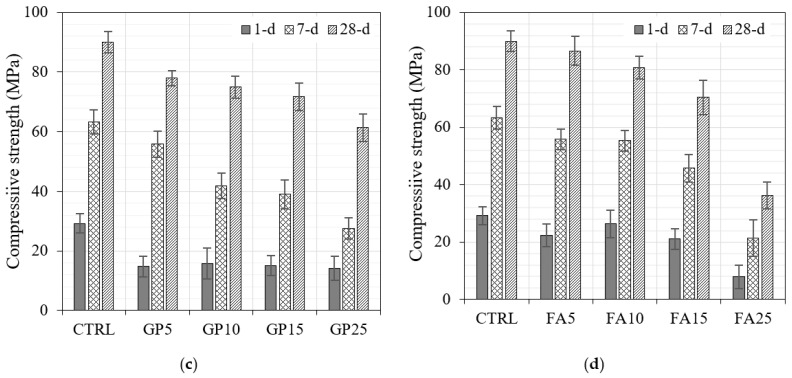
Compressive strength development of binary mixes (**a**) SF, (**b**) MK, (**c**) GP, and (**d**) FA.

The development of the compressive strength at 1, 7, and 28 days for the binary mixes is shown in Figure 15. The compressive strength reduced with replacement level with all powders. The strength was the highest in the mixes with SF in the following order: SF > MK > GP > FA. The reduction in strength due to the replacement level of SF is attributed to the reduction in workability and to the facts that the optimum replacement level of SF is around 5% and any further addition dilutes the cement content despite its high pozzolanic activity. Similarly, the incorporation of MK reduced the workability significantly, which reduced the strength. GP is much more reactive than FA; they initially behaved similarly, but at a high replacement level of 25%, GP maintained its activity, unlike FA. The reduction in the mix with 25% FA was the highest.

## 4. Conclusions

From the results obtained in this study, the following can be concluded:

- The individual performance of each type of supplementary cementitious material relies on its physicochemical properties and morphological structure, which affect both the fresh and hardened properties. Therefore, during the formulation of low-carbon concrete, the individual performance properties should be well identified to help in the development of ternary and quaternary mixes.
- The effect of SF and MK on the plastic viscosity can be compensated using FA, GP, or both.
- Both 5% SF and 5% MK provided similar levels of heat of hydration, while the incorporation of 5% GP led to a 10% reduction in the heat of hydration, and that of 25% FA reached 23% less than that of the control.
- The presence of MK increased the content of hydrated lime, while the incorporation of 5% SF led to an estimated reduction of about 3.8% in CH compared to the control mix. Therefore, with the incorporation of MK, it is preferable to add a material like GP, which has both high pozzolanic activity and a reducing effect on the plastic viscosity. GP can be identified in this case as a performance enhancer in the presence of MK.
- The highest pore area was found in the mix with 25% FA because of the higher content of pore water, followed by the MK5, GP5, and SF5 mixes and the control. Therefore, it is preferable to add SF along with FA to overcome this issue.
- The presence of FA shifted the temperature profile to a lower temperature and a longer time as an indication of the slower pozzolanic activity that directly reduced the heat of hydration. The incorporation of GP led to an increase in the temperature profile because of its accelerating effect on hydration.
- The strength was the highest in the mixes with SF in the following order: SF > MK > GP > FA. The reduction in the mix with 25% FA was the highest.
- The optimal ternary mixes from the powders under study are recommended for further study and refinement based on the findings of this study and the concept of performance enhancers.

## Figures and Tables

**Table 1:** Chemical analysis of fine powders.

Oxides (%)	PC	FA	SF	MK	GP
SiO_2_	20.41	55.23	86.20	52.6	68.83
Al_2_O_3_	5.32	25.95	0.49	36.45	0.36
Fe_2_O_3_	4.1	10.17	3.79	6.42	0.08
CaO	64.14	1.32	2.19	0.38	0.34
MgO	0.71	0.31	1.31	0.19	4.14
SO_3_	2.44	0.18	0.74	0.04	0.24
Na_2_Oeq	0.1	0.86	2.80	0.35	20.45
L.O.I	2.18	5	2.48	1.62	5.45
D50 (μm)	11	11	0.23	9	10

**Table 2:** Physical properties of red sand (RS).

Physical Properties	
Bulk Specific Gravity (OD Basis)	2.64
Bulk Specific Gravity (SSD Basis)	2.65
Apparent Specific Gravity	2.67
Absorption (%)	0.30
Fineness modulus (range of 2.3–3.1)	2.67

**Table 3:** The cement percentages of the developed mixes with a sand/binder ratio of 1.5.

Code	Binder				
	PC	SF	FA	MK	GP
CTRL	100	0	0	0	0
05SF	95	5	0	0	0
10SF	90	10	0	0	0
15SF	85	15	0	0	0
05FA	95	0	5	0	0
10FA	90	0	10	0	0
15FA	85	0	15	0	0
25FA	75	0	25	0	0
05MK	95	0	0	5	0
10MK	90	0	0	10	0
15MK	85	0	0	15	0
5GP	95	0	0	0	5
10GP	90	0	0	0	10
15GP	85	0	0	0	15
25GP	75	0	0	0	25

**Table 4:** Heat of hydration of the binary cementitious systems investigated.

	Heat of Hydration (J/g)
CTRL	154
SF5	150
MK5	151
GP5	140
FA25	119

**Table 5:** Thermal analysis and phase calculation after removing CO_2_ contribution to thermal loss.

	Total Loss (%) 0–1000 °C	CH (%) 350–450 °C	AFt (%) 100–200 °C	Calcite (%) 600–750 °C	Porewater (%)	Combined Water (%)
CTRL	18.16	12.85	9.00	6.29	5.14	20.93
SF5	17.74	11.72	8.90	6.03	5.68	20.39
MK5	18.25	13.63	9.49	5.67	5.32	20.75
GP5	17.52	12.83	8.48	5.84	6.41	19.66
FA25	15.20	11.06	7.53	4.86	8.30	17.77 *

* Contains 2% additional combined water, based on the theoretical calculation of total water of about 26%, because of the reaction with FA.

**Table 6:** Intrusion data summary of tested paste mixes.

	CTRL	SF5	MK5	GP5	FA25
Total pore area (m^2^/g)	15.48	15.71	16.85	16.28	19.65
Median pore diameter (volume) (nm)	60.6	67.3	60.8	62.7	58.1
Median pore diameter (area) (nm)	18.1	17.3	16.5	17.5	15.2
Average pore diameter (4V/A) (nm)	31.2	32.9	30.6	31.0	28.8

## Data Availability

Data are contained within the article.
